# Effects of Pre-Operative Risk Factors on Intensive Care Unit Length of Stay (ICU-LOS) in Major Oral and Maxillofacial Cancer Surgery

**DOI:** 10.3390/cancers13163937

**Published:** 2021-08-04

**Authors:** Juergen Wallner, Michael Schwaiger, Sarah-Jayne Edmondson, Irene Mischak, Jan Egger, Matthias Feichtinger, Wolfgang Zemann, Mauro Pau

**Affiliations:** 1 Department of Oral & Maxillofacial Surgery, Medical University of Graz, 8036 Graz, Austria; j.wallner@medunigraz.at (J.W.); egger@tugraz.at (J.E.); matthias.feichtinger@uniklinikum.kages.at (M.F.); Wolfgang.zemann@medunigraz.at (W.Z.); mauro.pau@uniklinikum.kages.at (M.P.); 2 Department of Cranio-Maxillofacial Surgery, AZ Monica and the University Hospital Antwerp, 2018 Antwerp, Belgium; 3 Department of Plastic and Reconstructive Surgery, Guy’s and St. Thomas’ Hospital, London SE1 7EH, UK; Sarah.edmondson4@nhs.net; 4 University Clinic of Dental Medicine and Oral Health, Medical University of Graz, 8036 Graz, Austria; irene.mischak@medunigraz.at; 5 Institute for Computer Graphics and Vision, Graz University of Technology, 8036 Graz, Austria

**Keywords:** risk factors, length of stay, ICU-LOS, oral cancer, microvascular, free flap reconstruction

## Abstract

**Simple Summary:**

This study aimed to investigate the effect of certain pre-operative parameters from the clinical routine directly on the post-operative intensive care unit (ICU)-length of stay (LOS) after major oral and maxillofacial cancer surgery. This study was performed to identify at-risk patients that are expected to need prolonged specialized care management post-operatively to these aforementioned operations. A homogenous cohort of 122 patients over a five year period was included in this study. At-risk patients are prone to need a significantly longer specialized care management than others. These patients are those with pre-operative severe renal dysfunction, peripheral vascular diseases and/or increasing heart failure stage categories. Confounding parameters that contribute to a prolonged specialized post-operative management in combination with other variables were identified as higher age, prolonged operative time, chronic obstructive pulmonary disease, and intra-operatively transfused blood.

**Abstract:**

Objective: This study aimed to investigate the effect of certain pre-operative parameters directly on the post-operative intensive care unit (ICU)-length of stay (LOS), in order to identify at-risk patients that are expected to need prolonged intensive care management post-operatively. Material and Methods: Retrospectively, patients managed in an ICU after undergoing major oral and maxillofacial surgery were analyzed. Inclusion criteria entailed: age 18–90 years, major primary oral cancer surgery including tumor resection, neck dissection and microvascular free flap reconstruction, minimum operation time of 8 h. Exclusion criteria were: benign/borderline tumors, primary radiation, other defect reconstruction than microvascular, treatment at other centers. Separate parameters used within the clinical routine were set in correlation with ICU-LOS, by applying single testing calculations (*t*-tests, variance analysis, correlation coefficients, effect sizes) and a valid univariate linear regression model. The primary outcome of interest was ICU-LOS. Results: This study included a homogenous cohort of 122 patients. Mean surgery time was 11.4 (±2.2) h, mean ICU-LOS was 3.6 (±2.6) days. Patients with pre-operative renal dysfunction (*p* < 0.001), peripheral vascular disease-PVD (*p* = 0.01), increasing heart failure-NYHA stage categories (*p* = 0.009) and higher-grade categories of post-operative complications (*p* = 0.023) were identified as at-risk patients for a significantly prolonged post-operative ICU-LOS. Conclusions: At-risk patients are prone to need a significantly longer ICU-LOS than others. These patients are those with pre-operative severe renal dysfunction, PVD and/or high NYHA stage categories. Confounding parameters that contribute to a prolonged ICU-LOS in combination with other variables were identified as higher age, prolonged operative time, chronic obstructive pulmonary disease, and intra-operatively transfused blood.

## 1. Introduction

The surgical treatment of advanced oral cancer often requires extensive resections in the head and neck area and the oral cavity [1,2,3]. In such cases microvascular free flaps are the gold standard treatment of choice for defect reconstruction, with an overall flap survival rate of approximately 90–95% [4,5,6].

Major oral cancer surgery with free flap reconstruction can be complex, highly invasive, lengthy, and exhausting [3,7] and, therefore, may necessitate prolonged invasive ventilation, post-operative cardio-pulmonary monitoring and/or sedation [8,9].

Although optimal measures for post-operative care following head and neck free flap reconstructions have already been discussed in the literature, and current trends are moving from routine ICU admission to immediate specialty unit recovery, the post-operative ICU admission rates and requirements still vary from one clinical center to another and, even within clinical institutions [10,11]. In this regard, it was shown that post-operative patient management in these complex head and neck cancer surgery patients can be performed safely in non-ICU specialty wards [12], however, that the majority of reconstructive surgeons will still send their patients to the ICU for the immediate post-operative period [11].

If the patient is admitted to an ICU post-operatively the ICU management hours or length of stay (LOS), which is the time until the patient is discharged from ICU, is a medically, as well as an economically relevant factor for both the patient and the clinical center [13,14,15,16]. For that reason, several studies have already focused on ICU-LOS and overall hospital LOS regarding specific advantages and disadvantages of ICU vs. non-ICU specialty wards, different sedation protocols, risk factors for post-operative complications, the effects of ICU staffing, the incidence of post-operative delirium and complex co-morbidity and mortality scores [9,11,17,18,19,20,21,22,23].

However, so far only a few studies have directly investigated the effects of separate pre-operative parameters on the post-operative ICU-LOS in major oral cancer surgery, independently from the already well-known incidence of post-operative delirium or anesthesiologic co-morbidity and mortality scores, that summarize many separate parameters to one single number and, are usually not determined within the routine pre-operative clinical assessment.

Therefore, the aim of this study was to identify separate prognostic parameters within routine pre-operative clinical assessment that are expected to relevantly prolong the patient’s post-operative intensive care management in those patients undergoing major oral and maxillofacial cancer surgery with free flap reconstruction. Pre-operatively, these parameters might be able to be used as reliable and independent indicators for a prolonged ICU-LOS within standard clinical practice, independently from the associated occurrence of post-operative delirium. The null hypothesis of this study pertains to that certain pre-operative parameters/risk factors do not directly significantly correlate with the post-operative ICU-LOS.

## 2. Material and Methods

### 2.1. Patients

This study was carried out in accordance with the local legal requirements and the Declaration of Helsinki (1975) at a tertiary clinical center (Medical University of Graz, Graz, Austria) and included the approval of the Ethics Committee of the University. Informed consent was obtained from all subjects before treatment. All data were deidentified before usage and stored in a protected Microsoft Excel^TM^ database.

A retrospective electronic clinical chart review was performed including surgical, anesthesiologic, and labor-chemical protocols, over a 5-year enrollment period (2015–2020), for data collection according to specific inclusion and exclusion criteria. All documentation in the analyzed electronic charts used for this study was performed by specialists such as oral and maxillofacial surgeons, anesthesiologists, pharmacists, and trained nurses.

In this study only, patients undergoing post-operative ICU admission following surgery were considered for inclusion and participation.

Inclusion criteria entailed: age over 18, age under 90, malignant tumor (oral cancer), major primary oral cancer surgery including complete tumor resection, primary neck dissection and primary microvascular free flap reconstruction, with a minimum operative time of 8 h.

Exclusion criteria included: age under 18, age over 90, benign or borderline tumor, primary radiation, defect reconstruction other than with microvascular free flaps, second or third malignant tumors at the time of diagnosis, primary treatment at other clinical centers. Patients with post-operative management carried out in a high-intensity nursing ward, intermediate care unit, or other non-ICU specialty wards were excluded.

### 2.2. Surgical Procedure

Patients undergoing microvascular free flap reconstruction following major oral and maxillofacial cancer surgical resection in one single procedure were eligible for inclusion in this study. Patients undergoing local or regional flap reconstruction, temporary obturator insertion, or any other type of reconstruction aside from microvascular free flaps, were excluded from the study cohort, as in our practice, ICU management for the immediate postoperative period is rarely indicated.

All cases were surgically treated at our department in Graz, Austria, and were admitted to our ICU for the immediate post-operative period.

Patients were considered for ICU admission for the immediate post-operative period in the following cases:

(1) In the absence of appropriate intermediate specialized or step-down units, or high nurse-to-patient ratio on the ward (recommended nurse-to-patient ratios of 1:2 or 1:1);
(2) The co-morbidities, risk factors, and functional organ impairments that were present in many patients of the cohort; and,
(3) The decision of the operating surgeon.

These reasons and indications for a potential post-operative ICU consideration can also be found in the literature [10,16,22,24,25,26].

Post-operatively, patients were admitted to either an “open” or “closed” ICU, dependent on their co-morbidities, risk factors, ASA scores, and intraoperative health condition [10,19,22]. Although both of these models are designated as ICUs, the closed staffing model designates a critical care physician or team of providers for the management or co-management of the admitted patients. The open staffing model is known as a “low intensity” ICU that can be compared to other specialized intermediate care units in terms of patient management.

### 2.3. Post-Operative Management

Post-operatively, appropriate trained nursing staff-to-patient ratios, along with close medical support, standard flap monitoring, as well as airway and wound care management, were provided to all patients. If appropriate, anesthesiologic medication and substitutions (narcotics and others) were administered following anesthesiologic standards and protocols to reduce post-operative delirium rate. Sedation and ventilation assistance were only carried out if needed, dependent on the patient’s specific post-operative cardio-pulmonary functional requirements and post-operative delirium and others [10,20,21,22,23,27].

ICU rooms were equipped with suction regulators and patients were monitored continuously without interruptions. The majority of ICU staffing had a background in the management of post-operative oral cancer patients including post-operative free flap assessment. Nursing ratios during this study were 1:1 or 1:2. Post-operative free flap assessment was performed every 3 h within the first 24 h post-operatively and, then every 6 h until day three post-operatively.

All patients included in this study received Propofol and Remifentanil whilst ventilated. Feeding tube nutrition (nasogastric tube) was initiated on day one after surgery in all patients. Attempted ventilator wean was commenced for all patients on post-operative day one. Post-operative sedation was continued up until the point at which the following criteria permitted weaning: hemodynamic stability, absent need for inotropic support, sufficient gas exchange by spontaneous ventilation, normal blood pH, normal core body temperature and, normal post-operative course from a surgical point of view. The decision to discharge the patient from ICU was made by concurrent agreement with at least two medical specialists (anesthesiologist and oral-maxillofacial surgeon).

The postoperative ICU admissions in this study followed the already above given indications and reasons. Apart from the close medical support of a critical care physician, the administration of certain anesthesiologic medications and, the assisted ventilation and sedation, most of the post-operative care, we believe, could have been provided in specialized intermediate care, step down units, or high nurse-to-patient ratio wards appropriate for major head and neck surgery patients.

### 2.4. Analyzed Parameters

Demographic and surgical data of patients were compared to ICU-LOS. Analyzed demographic and operative parameters/variables included patients’: sex; age; primary tumor location/site; body mass index (BMI); performance status (American Society of Anesthesiology (ASA) classification); extent of heart failure (New York Heart Association (NYHA) classification); tumor size (TNM classification); type of neck dissection (uni-, bi-lateral); and, length of surgery (skin incision to end of wound closure).

The pre-operative existence (dichotic assessment) of the following patient factors were also analyzed: arterial hypertension, diabetes mellitus, adiposity (BMI > 30 kg/m^2^), coronary artery disease (CAD), renal dysfunction (GFR < 60 mL/min), chronic alcoholism (estimated daily ethanol intake of >200 mL), chronic smoking (>10 cigarettes daily for at least 3 years), chronic obstructive pulmonary disease (COPD), and peripheral vascular disease (PVD).

**Figure 1:**
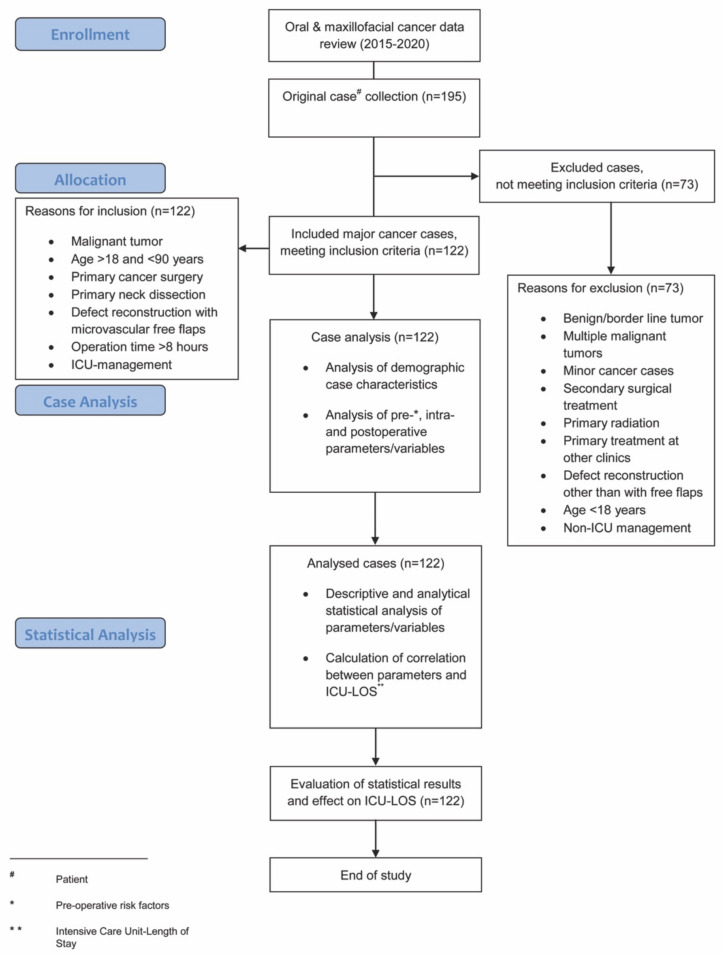
Flowchart diagram demonstrating the steps of the study protocol.

Analyzed intra-operative parameters included: estimated blood loss, amount of intra-operative blood transfused (erythrocyte concentrates during operation), urinary output, hemoglobin level, and maximum temperature. Analyzed post-operative parameters included ICU-LOS and post-operative complications, as per the Clavien–Dindo classification [28]. This internationally well-established classification categorizes post-operative complications with a high level of validity and reliability using grades I-IV and was amongst others recently comparably used in the works of Pau et al., and Rempel et al., regarding post-operative complications in squamous cell carcinoma surgery [3,29]. A flowchart diagram demonstrates the steps of the study protocol (Figure 1).

### 2.5. Statistical Analysis

The analyzed primary outcome parameter was ICU-LOS (hours). Analyzed demographic and pre-operative parameters (variables) were set in correlation with ICU-LOS using analytical statistical methods. This also included the amount of intra-operative blood transfusions and any post-operative complications. Descriptive statistical methods were used for the analyzed parameters, blood loss during reconstruction (estimated), urinary output, hemoglobin level, maximum temperature, and primary tumor site. These variables were not set in correlation with ICU-LOS. An intention to treat analysis was used when calculating the results of this study.

A univariate general linear model of regression, including *p*-value calculation, was conducted to assess the correlation of several variables with the primary outcome of interest, ICU-LOS. The aforementioned regression analysis was used to test for unadjusted associations between ICU-LOS and potential risk factors and to adjust for potential confounding by the variety of co-variables. The regression analysis included the calculation of the quality value R^2^ (0–1), which gives the correlation between observed and predicted (calculated) values, in order to prove the regression model’s validity.

The univariate general linear model of regression calculates the association of several pre-operative patient risk factors and parameters (variables) with the primary outcome of interest, ICU-LOS. The regression analysis was used to test for unadjusted associations between ICU-LOS and potential risk factors and to adjust for potential confounding by the variety of co-variables. Prior to regression analysis, single testing of differences amongst the variables and the numerical primary outcome, ICU-LOS, was performed using correlation coefficients (Pearson, Spearman), the analysis of variance (ANOVA), or *t*-test calculations for numerical, categorical, and dichotomous variables when appropriate. The results were corrected for multiple testing. Effect sizes were given as Cohen’s d (*t*-tests) and Eta Square-η^2^ (ANOVA). However, single testing calculations only focus on the effect of a single parameter without considering any additional effect of other co-variables that may also influence ICU-LOS in parallel.

For all calculations a *p*-value of *p* < 0.05 was considered statistically significant (95% confidence intervals of differences were given for *t*-tests). The data gathered from these calculations are presented using descriptive and analytical statistical methods such as means ± standard deviations (SD) unless otherwise stated.

All statistical analyses were performed using the open-source statistical package R version 3.5.1 (© 2019 The R-Foundation for Statistical Computing, http://www.r-project.org, accessed on 1 April 2021).

## 3. Results

**Figure 2:**
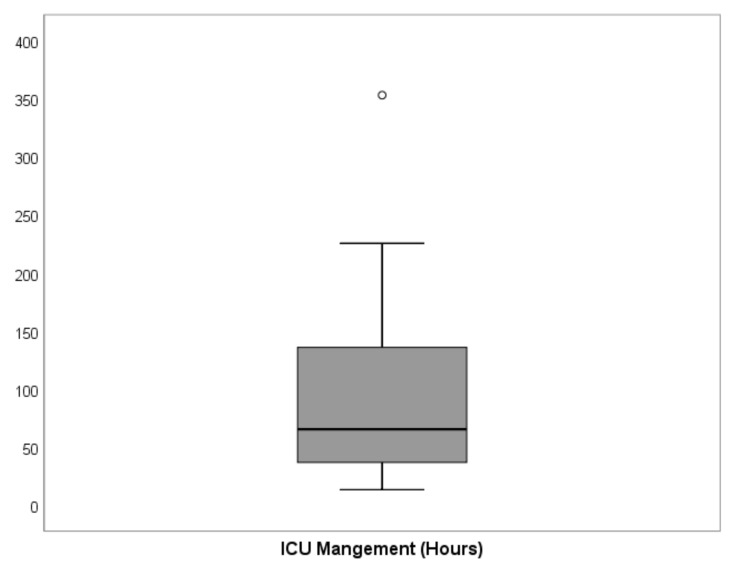
Graphical distribution of the primary outcome, ICU-LOS for all cases (*n* = 122). The black bold line indicates the median, while the boxes show the interquartile range (IQR) between 25th and 75th percentile and whiskers indicate the minimum and maximum values within 1.5 × IQR. One extreme value was observed. The detailed values of ICU-LOS are given in Table 2.

**Figure 3:**
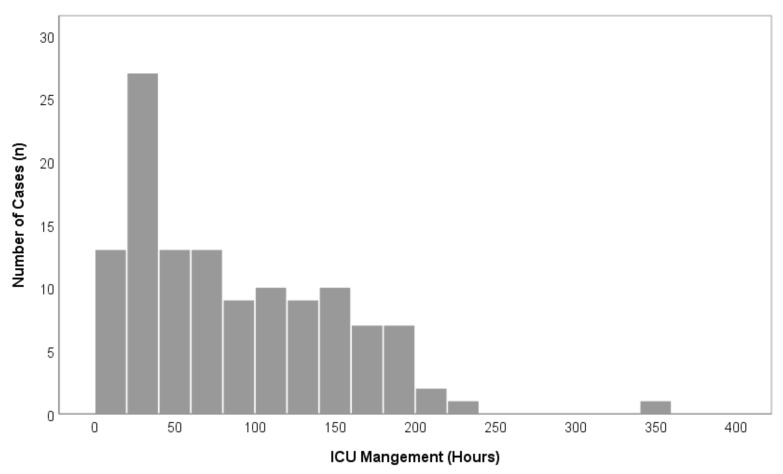
Overview of overall case distributions in comparison to ICU-LOS (*n* = 122).

A total of 195 patients were treated with oral and maxillofacial cancer at our department during the recruitment period. Of these, 122 patients (76 men, 46 women) underwent major surgical oral cancer resection, with subsequent free flap reconstruction and met the inclusion criteria. A total of 73 patients did not meet the inclusion criteria and were excluded from this study (Figure 1). Within the study population, the mean age was 61.5 (±10.0) years (Table 1). The floor of the mouth was the most common primary tumor site (28.7%), followed by the oropharynx (18.9%), the mandible (18.0%), and the tongue (15.6%). A total of 54.9% of cases underwent bilateral neck dissection and in 45.1% unilateral neck dissection was performed (Table 1). The parameters blood loss, urinary output, hemoglobin level, and maximum temperature after surgery are given in Table 2. The average ICU-LOS was 87.0 (±63.3) h which equates to approximately 3.6 (±2.6) days (Figure 2 and Figure 3), (Table 3). No differences regarding ICU-LOS between men (85.5 ± 61.5 h) and women (89.5 ± 66.8 h) were observed. The average operative time was 11.4 (±2.2) h (Table 4). No adverse events during ICU management or at ICU discharge were observed unless mentioned within the post-operative complication grade categories.

The univariate general linear model of regression showed significant values (*p* < 0.05) for the variables BMI, renal dysfunction, the extent of heart failure (NYHA classification Grade I-III), PVD, and post-operative complications (Clavien-Dindo classification Grade 0-III) (Table 4).

**Figure 4:**
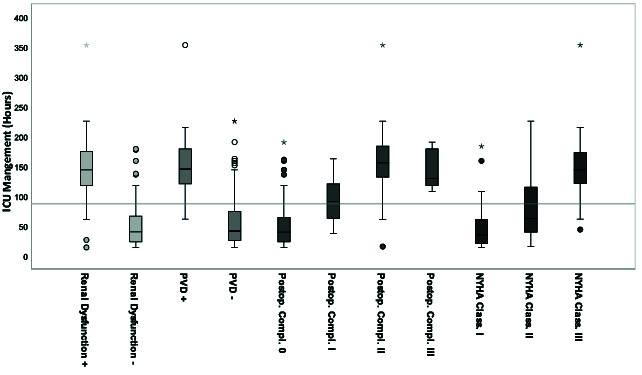
Pre-operative significant parameters in correlation to ICU-LOS (*n* = 122) according to the calculated univariate general linear regression model. The grey line marks the mean ICU-LOS, 87 (±63.3) h, 3.6 days. Each significant parameter is given as standard boxplot diagram including the marked median, the interquartile range (IQR), the 1.5xIQR and the extreme values. Extreme values are marked as circles (1.5-3xIQR) and stars (>3xIQR). (ICU: Intensice Care Unit, PVD: Peripheral Vascular Disease, NYHA Class.: Extent of Heart Failure according to the New York Heart Association Grade Classification, Postop. Compl.: Postoperative Complication according to the Clavien-Dindo Classification Grades, + positive, - negative).

According to the calculated regression analysis, these values correlated significantly with ICU-LOS, although BMI was only weakly significant with low correlation (Pearson: r = 0.010) and without significance with high BMI values <30 kg/m^2^ (adiposity). Mean ICU-LOS was increased by approximately 66% in cases with positive renal dysfunction, by 74% in cases with positive PVD, by 71% in cases with NYHA stage III, and by 82% and 64% in cases with Clavien-Dindo stage II and stage III, respectively. An increasing NYHA stage by one grade category resulted in a corresponding 55% increase in ICU-LOS. An increasing post-operative complication stage by one grade category resulted in a corresponding approximately 57% increase in ICU-LOS between the grades 0, I, and II (Figure 4).

The majority of post-operative complications were treated conservatively using pharmacological treatments, except in nine cases (7.4%) where secondary surgical treatments (cessation of bleeding, revision of anastomosis, etc.) were performed. Complete flap loss was observed in three cases (2.5%) and in these cases, a second free flap was harvested immediately. No life-threatening post-operative complications (grade category IV) were observed. No death during hospitalization occurred.

Increasing grade categories that resulted in a corresponding increase in ICU-LOS were also analyzed for the requirement of blood transfusion (erythrocyte concentrates), tumor size (T-classification), and the patient’s performance status (ASA classification), however, none were significant (Table 4).

The highest correlation with ICU-LOS (*p* < 0.01) was observed in the parameters “renal dysfunction” (GFR < 60 mL/min) and the extent of heart failure (NYHA classification) (Table 4). The regression analysis included the quality value R^2^ (0–1) = 74%, which confirms the validity of the used regression model, meaning that the performed calculation values are at least valid for the main part of our patient cohort (74%).

The single testing of parameters was highly significant for the same parameters as in the regression model, except for the parameter BMI. All these parameters also showed high effect sizes (Cohen’s d or Eta Square η^2^) or high positive correlation coefficients (Table 4 and Table 5). The single testing of parameters was also significant for some other additional variables such as the patient’s age, length of surgery, tumor size, intra-operative blood transfusions, COPD, ASA performance status, and the type of neck dissection (Table 5). However, it should be noted that the single testing of parameters (*t*-tests, ANOVA) does not control multiple possible confounders by the variety of co-variables (sex, age, type of neck dissection, tumor localization, etc.). Single testing calculations (Table 5) only focus on the effect of a single parameter without considering any additional effect of other variables that may also influence ICU-LOS in parallel. For that reason, the variety of multiple possible confounders that may additionally influence ICU-LOS in parallel were considered in a separate regression model (Table 4).

## 4. Discussion

Advanced head, neck, and oral cancer surgery with free flap reconstruction often necessitate patient admission to ICU post-operatively or, at the very least, a step-down/intermediate care unit, admission [11]. The high clinical relevance of identifying prognostic parameters for a prolonged ICU-LOS has repeatedly been shown in the literature [9,10,19,29].

It has to be highlighted that post-operative ICU admission is not the standard of care at many centers. Against this backdrop, the present study does not want to question guidelines or recommendations concerning the peri-operative patient care in head and neck surgery. However, clinically appropriate post-operative patient management still varies widely between centers [10], and a defined consensus, of specific criteria to determine the need for post-operative ICU versus non-ICU admission, especially in at-risk patients remains incomplete [22].

ICU admission depends upon the patient’s pre-operative comorbid status, the intra-operative complication rates, the reconstructive surgeon’s preference, and potentially also on the human and financial resources of the clinical center [10,18,22,29,30]. Regarding the postoperative care in major head and neck cancer patients, it was shown that in patients with pre-operatively present co-morbidities, high ASA grades, cardio-pulmonary or renal functional impairments and/or other risk factors a post-operative ICU admission can be considered [16,25,26].

In our study cohort, many patients with such aforementioned risk factors were present. What is more, bilateral neck dissection, blood transfusions, treatment of postoperative complications, heavy smoking histories, and other preoperatively existing co-morbidities were observed within the selected patient cohort of our study. In the literature, many studies suggest the consideration of postoperative ICU admission for the immediate postoperative period as appropriate patient care in major head and neck surgery when such aforementioned risk factors are present [16,25,26]. Therefore, such major cases, including extensive tumor resection and complex free flap reconstruction, as discussed in this study, may practically more likely be admitted to the ICU for the immediate post-operative period than others.

ICU-LOS is both a highly relevant medical and economic factor, which correlates with an increased incidence of pneumonia, use of narcotics, and also with increased health costs and staffing resources [9,10,19,31]. This study, therefore, aimed to identify those at-risk patients from their pre-operative status, who could be predicted to require a prolonged intensive care management period following major oral cancer surgery, in order to potentially improve and adjust those at-risk parameters.

When analyzing the cohort of this study, we found that one-third of patients had their primary tumor site located in the floor or anterior base of the mouth. A similar distribution was also described by Sundermann et al., in a large-scale evaluation of oral cavity carcinoma localization [32]. We also observed similarities in our study population regarding age, blood loss, and urinary output during surgery comparable to other reported cohorts in this field [19,20].

In this study, the mean ICU-LOS (~3.6 days) was found to be within a similar range compared to other published protocols, which indicate that most ICU-LOS admissions are between 24 and 72 h for the major head, neck, and maxillofacial patients with free flap reconstruction [5,6,9,13,33,34,35,36]. The duration of surgery was slightly higher in this study (~11.4 h) than in similar investigations, however, with a mean operation time of three hours more, it is still in a comparable range [29].

According to our results, several parameters were found to significantly prolong ICU-LOS. These were: (1) pre-operatively diagnosed PVD, (2) increasing grades of post-operative complications and especially, (3) the presence of relevant renal dysfunction, and (4) increasing NYHA grade categories. These parameters showed statistically significant results in both the regression model and the single testing calculations, including high effect sizes and positive correlation coefficients. Thus, the null hypothesis of this study can be rejected and the alternative hypothesis can be confirmed, stating that pre-operative risk factors including increasing grades of post-operative complications, directly affect the length of ICU management after advanced oral cancer surgery with free flap reconstruction. Additional to these parameters, associated concurrent postoperative delirium was not seen as a systematic bias mainly responsible for a significant prolonged ICU-LOS.

This is due to anesthesiologic medication and substitutions (narcotics and others) only being administered according to anesthesiologic standards and protocols to minimize and limit the delirium rate.

Some other variables also showed significance. Increased BMI correlated with significance in the regression model and, the patient’s age, length of surgery, tumor size, intra-operative blood transfusions, COPD, the ASA performance status, and type of neck dissection were significant in the single testing calculations.

In this context, a higher amount of intra-operatively given blood transfusion was also associated with a prolonged ICU-LOS by Rempel et al., and the parameter age was already found to be an independent factor for prolonged ICU-LOS by Kesting et al., in head and neck carcinoma surgery [37]. However, the significant effect of BMI on ICU-LOS may be negated, as this parameter showed a nearly non-existent correlation towards zero and, in addition, very high BMI values (adiposity) were not found to be significant. Furthermore, only moderate correlations were found for the parameters “tumor size” and “age” and the weak effect size was found for the parameter “ASA performance status”.

Parameters, that were only significant in the single testing calculations and not in the regression model, were assessed as confounding variables in this study. This was justified by the missing adjustment for potential confounding variables (confounders) by the variety of co-variants; which is the case when only single testing calculations such as *t*-test or ANOVA are employed, without using an additional regression model. For that reason, single testing calculations alone cannot be reliably correlated with ICU-LOS and significant single tested parameters should, therefore, be additionally assessed by taking a valid regression model analysis into account.

Increasing patient age and prolonged operative length have already been established as risk factors for the occurrence of post-operative delirium. In addition, increasing patient age and tumor size were identified as parameters that correlate significantly with increased ICU-LOS, following head and neck free flap reconstruction [20,21,38]. According to our results, the aforementioned parameters, especially age over 70, were found to correlate in this study with a prolonged ICU-LOS, however, more as confounders in combination with other variables, rather than as separate significant risk factors alone. These findings are in accordance with the results published by Bhama et al. [19].

In the literature, the identification of pre-operative risk factors in correlation with ICU-LOS has already been included in prospective investigations of ICU staffing effects, different sedation protocol impacts after head, neck or maxillofacial surgery [9,19] or the prognostic implication analysis of comorbidities [29] and complication rates [39] for days spent in the ICU. Therefore, this study is not built upon a new research topic. However, differently to other already existing studies, that also used ICU-LOS as a primary outcome of interest, this investigation did not widely summarize a variety of different pre-operative parameters to one single number by using simplified scores such as the Simplified Acute Physiology Score (SAPS II) [40], the Acute Physiology and Chronic Health Evaluation II (APACHE II) score [41] or the Charlson Comorbidity Index (CCI) [42].

The disadvantage of using such expanded co-morbidity scores is that through the summary of a wide range of parameters to one simplified index, a retrospective identification and analysis of pre-operatively defined relevant variables is difficult. Furthermore, such scores and indices are usually not determined within the routine pre-operative clinical assessment. They are more likely to be used as anesthesiologic tools to measure the overall severity of a patient’s disease or the overall mortality risk regarding ICU management [41,42]. For these reasons, we chose to focus on pre-operative parameters that are routinely assessed in every patient before surgery; as such, these parameters might be able to be used as reliable indicators for a prolonged ICU-LOS within standard clinical practice.

In all parameters that were identified to significantly correlate with ICU-LOS, mean ICU-LOS was increased by at least 60% (50 h or more). The highest correlation with ICU-LOS was observed in the parameters “renal dysfunction” (GFR < 60 mL/min) and the extent of heart failure (NYHA classification), meaning that these parameters most strongly affect ICU-LOS. This may be explained through the fact that relevant renal dysfunction leads to a decreased elimination rate of certain toxins in the blood plasma, creating multi-organ damage on a cellular level and further impairs cardio-pulmonary function. Unstable renal and cardio-pulmonary systems have both crucial consequences on the overall health condition of the patient and, increase the overall morbidity and mortality [43]. This may be especially true for the immediate post-operative period in major surgical cases.

It was reported that a prolonged ICU-LOS negatively affects the patient’s overall health condition and 5-year mortality [10,19], and in addition, prolonged ICU-LOS (of approximately 50 h) also results in an additional 5400. Euros financial health care cost per patient. This stands in accordance with other reports evaluating ICU hospital costs [9,31].

We are aware of some limitations of our study, which include: (1) the size of our study collective; (2) the single-center evaluation; (3) the use of specific clinical methods at our center that may influence study results and reduce outcome validity for others; (4) the missing inclusion data in non-ICU wards; (5) the missing evaluation of multiple reconstruction types; and, (6) the missing evaluated details and differences between “open” and “closed” ICUs.

The rationale behind these limitations is discussed now in detail:

(1) Although the literature provides studies with more cases, we tried to form a homogenous and representative cohort of patients over a 5-year period according to strict inclusion and exclusion criteria. Additionally, we used both single testing calculations and regression model analysis to provide valid correlation assessments between parameters and ICU-LOS.
(2) A multi-center evaluation was not performed at this stage, because post-operative treatment protocols may vary widely between centers and, it would be difficult to ensure consistent patient management within one study population. This is probably also the reason why most existing studies in this field use single-center designs.
(3) The ICU management of our study population was comparable to other reports in terms of sedation protocols, nurse-staffing ratios, or length of stay [9,19,33,34]. Age categories, the amount of blood loss, and urinary outputs during surgery were also similar to other cohorts in this field [19,20]. Furthermore, our treatment protocol followed the consensus and recommendations for optimal peri-operative care in major head and neck cancer surgery with free flap reconstruction [22]. Therefore, the results of this study should at least be partly valid for other institutions. Certainly, specific methods at our center may probably influence the study outcomes in some way, however, as this would probably also be true for other studies at other centers, we tried to decrease this limitation by evaluating patients over several years and creating a homogenous study cohort according to strictly defined criteria.
(4) Since the primary outcome parameter of this study was ICU-LOS, we focused on patients that were postoperatively admitted to ICU. We did not separately include patients managed at step-down or intermediate care units. However, analysis of parameters affecting the length of stay in these units is planned for future research.
(5) This study focused on defect reconstruction using microvascular free flaps. Other reconstruction types were not found to necessarily need to be transferred to the ICU for the immediate postoperative period. Since this work investigated the identification of prognostic parameters for a prolonged length of stay specifically in an ICU, those other reconstructions were excluded.
(6) Since all patients included in this study were admitted to an ICU for the immediate postoperative period, no further differences were made in our retrospective analysis between “open” and “closed” ICU admissions. This study included all patients that were submitted to an ICU, independently of whether the units were run under an “open” or “closed (low intensity)” strategy. The results of this study are therefore valid for both “open” and “closed” ICUs. Independently from that, most of the postoperative treatment that was carried out could probably also have been carried out in non-ICU wards. Therefore, the results of this study may also be extrapolated for non-ICUs, such as specialized intermediate care, step-down units, or high-intensity nursing wards.

## 5. Conclusions

At-risk patients are prone to needing significantly longer ICU-management periods than others. These patients are those with pre-operative severe renal dysfunction, PVD, and/or high NYHA stage categories. Confounding parameters that contribute to a prolonged ICU-LOS in combination with other variables were identified as higher age, prolonged operative time, COPD, and intra-operatively transfused blood.

The early-stage identification of relevant risk factors in all patients scheduled for major oral, head and neck, and maxillofacial cancer resection, before surgery, can decrease the post-operative management period spent in ICU and, may improve the general pre- and post-operative health condition of the patient, the overall 5-year mortality rate and the overall therapy status outcome.

Pre-operative initiation of risk factor optimization at the earliest possible time point, and the concurrent present health issue compensation, following identification of potential predictors for a prolonged post-operative management period, is essential: it is even more important than during or after surgery.

Especially in at-risk patients, the presence of close medical support and appropriately trained nurse staff ratios (patient-to-nurse 1:1, or at least 1:2) should be ensured, more so than in not-at-risk patients, due to an expected prolonged need and complexity of immediate postoperative care. In the absence of specialized intermediate care, step-down units or high-nursing wards, these at-risk patients can be considered for admission to an “open” (low intensity) ICU for the immediate postoperative period. This will ensure safe and appropriate patient care after major oral, head and neck, and maxillofacial surgery, with adjuvant complex free flap reconstruction.

## Figures and Tables

**Table 1:** Categorical distribution of cases (*n* = 122) (SD: Standard Deviation) and distribution of primary tumor site and neck dissection (*n* = 122) (Maxilla: Maxilla/Upper Jaw/Upper Alveolar Ridge, Mandible: Mandible/Lower Jaw/Lower Alveolar Ridge, Other: Lip/Cheek/other Oral Cavity, BLND: Bilateral Neck Dissection, ULND: Unilateral Neck Dissection).

**Sex**	***n* (%)**	**Mean Age (±SD)**
Male	76 (62.3)	61.9 (±9.6)
Female	46 (37.7)	60.9 (±10.6)
Overall	122 (100)	61.5 (±10.0)
**Primary Tumor Site**	***n* (%)**	
Floor of Mouth	35 (28.7)	
Mandible	22 (18.0)	
Maxilla	11 (9.0)	
Oropharynx	23 (18.9)	
Other	11 (9.0)	
Tongue	19 (15.6)	
Tonsil	1 (0.8)	
**Primary Site of Neck Dissection**	***n* (%)**	
BLND	67 (54.9)	
ULND	55 (45.1)	

**Table 2:** Numerical analysis of intra-operative parameters (*n* = 122) (mL: Milliliter, g/dL: gram per deciliter, °C: Celsius Degree, Min: Minimum, Max: Maximum). Note: Hemoglobin level and maximum temperature were analyzed at the end of surgery.

Intra-Operative Parameters	Min	Max	Mean	SD (±)
Blood Loss (estimated) (mL)	260	670	445.3	±96.4
Hemoglobin Level (g/dL)	7.8	13.1	10.1	±1.4
Urinary Output (mL)	430	1730	989.5	±307.6
Maximum Temperature (°C)	37	39	38.1	±0.5

**Table 3:** Overall distribution of ICU-LOS (*n* = 122) (SD: Standard Deviation).

Primary Outcome Parameter	Min	Max	Median	Mean	SD (±)
ICU-LOS (h)	13.5	353.0	65.5	87.0	±63.3

**Table 4:** Numerical and categorical analysis of pre-operative parameters in correlation to ICU-LOS given in hours (*n* = 122). The table shows a univariate general linear model of regression that was conducted to assess the correlation of several variables with the primary outcome of interest, ICU-LOS. This regression model calculates the association of several pre-operative patient risk factors and parameters (variables) with the primary outcome of interest, ICU-LOS, by considering the variety of multiple possible confounders that may additionally influence ICU-LOS in parallel. The table also includes the analysis of post-operative complications. *p*-values of <0.05 were considered statistically significant. The correlation between observed and predicted (calculated) values (proof of regression model’s validity) was calculated with R^2^ (0–1) = 0.739 (~74%).

**Parameter**	**Min**	**Max**		**Mean (±SD)**	***p*-Value**
Age (years)	42	82		61.5 (±10.0)	*p* = 0.879
BMI (kg/m^2^)	17.5	40.1		27.0 (±5.2)	*p* = 0.038
Time of Surgery (h)	8	16		11.4 (±2.2)	*p* = 0.312
**Parameter**		***n***	**%**	**Mean ICU-LOS (±SD)**	***p*-Value**
Sex	male	76	62.3	85.5 (±61.5)	*p* = 0.609
	female	46	37.7	89.5 (±66.8)	
ASA Performance Status	I	27	22.1	42.8 (±32.2)	*p* = 0.243
(Grade I-VI)	II	39	32.0	80.6 (±57.2)	
	III	55	45.1	113.8 (±66.5)	
	IV	1	0.8	62.5	
Arterial Hypertension	+	57	46.7	117.8 (±64.1)	*p* = 0.513
(positive +, negative −)	−	65	53.3	60.1 (±49.0)	
Diabetes Mellitus	+	20	16.4	78.3 (±62.1)	*p* = 0.248
(positive +, negative −)	−	102	83.6	88.8 (±63.7)	
Adiposity (BMI >30 kg/m^2^)	+	37	30.3	79.2 (±60.4)	*p* = 0.207
(positive +, negative −)	−	85	69.7	90.4 (±64.6)	
CAD	+	32	26.2	69.8 (±53.7)	*p* = 0.930
(positive +, negative −)	−	90	73.8	93.2 (±65.6)	
Renal Dysfunction	+	45	36.9	144.3 (±55.6)	*p* < 0.001
(GFR <60 mL/min)	−	77	63.1	53.6 (±38.9)	
Chronic Alcoholism	+	91	74.6	89.4 (±63.8)	*p* = 0.932
(estimated daily ethanol intake of >200 mL)	−	31	25.4	80.2 (±62.3)	
Chronic Smoking	+	104	85.2	84.8 (±63.8)	*p* = 0.725
(>10 cigarettes daily for at least 3 years)	−	18	14.8	100.1 (±60.3)	
COPD	+	35	28.7	115.9 (±57.9)	*p* = 0.239
	−	87	71.3	75.4 (±61.9)	
Extent of Heart Failure	I	48	39.3	45.5 (±35.9)	*p* = 0.009
(NYHA Classification	II	39	32.0	82.9 (±55.0)	
Grade I–IV)	III	35	28.7	148.6 (±52.4)	
PVD	+	37	30.3	151.5 (±50.6)	*p* = 0.010
(positive +, negative −)	−	85	69.7	59.0 (±45.3)	
Tumor Size	T1	9	7.4	58.3 (±110.7)	*p* = 0.214
(T-Classification, T1–T4)	T2	42	34.4	53.9 (±42.5)	
(UICC 2017)	T3	29	23.8	103.7 (±50.4)	
	T4	42	34.4	114.9 (±59.9)	
Blood Transfusion	0	67	54.9	51.7 (±39.9)	*p* = 0.603
(Number of intra-operative Erythrocyte	1	29	23.8	109.2 (±71.6)	
Concentrate–EC	2	24	19.7	155.0 (±30.9)	
1 EC = 345.3 mL suspension)	3	2	1.6	134.3 (±37.2)	
Post-operative Complication	0	73	59.8	52.8 (±39.4)	*p* = 0.023
(Clavien-Dindo Classification	I	13	10.7	93.2 (±40.0)	
Grade I–IV, 0 = normal)	II	27	22.1	158.1 (±60.5)	
	III	9	7.4	142.9 (±32.4)	
	IV	0	0	0	

(EC: Erythrocyte Concentrate; kg: Kilogramm, m: Meter, ASA: American Society of Anaesthesiology, NYHA: New York Heart Association, PVD: Peripheral Vascular Disease, COPD: Chronic Obstructive Pulmonary Disease, CAD: Chronic Artery Disease, BMI: Body Mass Index, GFR: Glomerular Filtration Rate, mL: Milliliter, min: Minute; SD: Standard Deviation, Min: Minimum, Max: Maximum).

**Table 5:** Separate single testing of parameters in correlation to ICU-LOS. The table shows the calculation of single parameters that were set in correlation to ICU-LOS separately. This calculation does not control multiple possible confounders from other co-variables such as sex, age, type of neck dissection, tumor localization etc. The variety of multiple possible confounders that may additionally influence ICU-LOS in parallel was considered in the regression model in Table 4. Mean values of analyzed parameters and values of ICU-LOS are also included in Table 4. The statistical methods used in this table are given in detail in the material and methods section of the manuscript.

Parameter	*p*-Value	Testing	Correlation Coefficient	Effect Size	95% CI ćof Diff
Age (years)	*p* < 0.001	Pearson	r = 0.587		
BMI (kg/m^2^)	*p* = 0.913	Pearson	r = 0.010		
Time of Surgery (hours)	*p* = 0.001	Pearson	r = 0.286		
Type of Neck Dissectionć (ULND, BLND)	*p* < 0.001	*t-*test		Cohens’d = 1.01	[36.7–77.6]
Sex	*p* = 0.735	*t*-test		Cohens’d = 0.06	[−27.5–19.5]
(men, women)					
ASA Performance Status	*p* < 0.001	ANOVA		η^2^ = 0.19	
(Grade I-VI)					
Arterial Hypertension	*p* < 0.001	*t*-test		Cohens’d = 1.01	[37.4–78.0]
Diabetes Mellitus	*p* = 0.501	*t-*test		Cohens’d = 0.17	[41.2–20.3]
Adiposity (BMI > 30 kg/m^2^)	*p* = 0.370	*t*-test		Cohens’d = 0.18	[−35.9–13.5]
CAD	*p* = 0.072	*t*-test		Cohens’d = 0.39	[−49.0–2.2]
Renal Dysfunction	*p* < 0.001	*t*-test		Cohens’d = 1.89	[73.7–107.7]
(GFR < 60 mL/min)					
Chronic Alcoholism	*p* = 0.488	*t*-test		Cohens’d = 0.15	[−16.9–35.3]
(estimated daily ethanol intake of >200 mL)					
Chronic Smoking	*p* = 0.345	*t*-test		Cohens’d = 0.25	[−16.9–35.3]
(>10 cigarettes daily for at least 30 years)					
COPD	*p* = 0.001	*t*-test		Cohens’d = 0.68	[−16.4–64.6]
Extent of Heart Failure	*p* < 0.001	ANOVA		η^2^ = 0.44	
NYHA Classification					
(Grade I-IV)					
PVD	*p* < 0.001	*t*-test		Cohens’d = 3.03	[74.2–110.8]
Tumor Size (Classification, T1-T4)	*p* < 0.001	Spearman	r = 0.487		
(UICC 2017)					
Blood Transfusion	*p* < 0.001	Spearman	r = 0.672		
(Number of intra-operative Erythrocyte)					
Concentrate–EC					
(1 EC = 345.3 mL suspension)					
Post-operative Complicationć (Clavien Dindo Classificationć Grade I-IV, 0 = normal)	*p* < 0.001	Spearman	r = 0.681		
			
			

EC: Erythrocyte Concentrate; BLND: Bilateral Neck Dissection, ULND: Unilateral Neck Dissection; kg: Kilogramm, m: Meter, ASA: American Society of Anaesthesiology, NYHA: New York Heart Association, PVD: Peripheral Vascular Disease, COPD: Chronic Obstructive Pulmonary Disease, CAD: Chronic Artery Disease, BMI: Body Mass Index, GFR: Glomerular Filtration Rate, mL: Milliliter, min: Minute; SD: Standard Deviation, Min: Minimum, Max: Maximum, CI of Diff: Confidence Intervall of Difference.

## Data Availability

The data presented in this study are available on request from the corresponding author. The data are not publicly available due to an ongoing research project which builds up on the calculations and results of in this work.
